# Myc and Fgf Are Required for Zebrafish Neuromast Hair Cell Regeneration

**DOI:** 10.1371/journal.pone.0157768

**Published:** 2016-06-28

**Authors:** Sang Goo Lee, Mingqian Huang, Nikolaus D. Obholzer, Shan Sun, Wenyan Li, Marco Petrillo, Pu Dai, Yi Zhou, Douglas A. Cotanche, Sean G. Megason, Huawei Li, Zheng-Yi Chen

**Affiliations:** 1 Department of Otolaryngology, Harvard Medical School, Boston, Massachusetts, United States of America; 2 Eaton-Peabody Laboratory, Massachusetts Eye and Ear Infirmary, Boston, Massachusetts, United States of America; 3 Department of Systems Biology, Harvard Medical School, Boston, Massachusetts, United States of America; 4 Department of Otorhinolaryngology, Shanghai Eye and ENT Hospital, Shanghai Medical College, Fudan University, Shanghai, China; 5 Department of Otolaryngology, Chinese PLA General Hospital, Beijing, China; 6 Stem Cell Program and Division of Pediatric Hematology/Oncology, Children’s Hospital Boston and Dana-Farber Cancer Institute, Boston, Massachusetts, United States of America; 7 Cell and Developmental Biology, University of Massachusetts Medical School, Worcester, Massachusetts, United States of America; University of Washington, Institute for Stem Cells and Regenerative Medicine, UNITED STATES

## Abstract

Unlike mammals, the non-mammalian vertebrate inner ear can regenerate the sensory cells, hair cells, either spontaneously or through induction after hair cell loss, leading to hearing recovery. The mechanisms underlying the regeneration are poorly understood. By microarray analysis on a chick model, we show that chick hair cell regeneration involves the activation of proliferation genes and downregulation of differentiation genes. Both *MYC* and *FGF* are activated in chick hair cell regeneration. Using a zebrafish lateral line neuromast hair cell regeneration model, we show that the specific inhibition of Myc or Fgf suppresses hair cell regeneration, demonstrating that both pathways are essential to the process. Rapid upregulation of Myc and delayed Fgf activation during regeneration suggest a role of Myc in proliferation and Fgf in differentiation. The dorsal-ventral pattern of *fgfr1a* in the neuromasts overlaps with the distribution of hair cell precursors. By laser ablation, we show that the *fgfr1a*-positive supporting cells are likely the hair cell precursors that directly give rise to new hair cells; whereas the anterior-posterior *fgfr1a*-negative supporting cells have heightened proliferation capacity, likely to serve as more primitive progenitor cells to replenish lost precursors after hair cell loss. Thus *fgfr1a* is likely to mark compartmentalized supporting cell subtypes with different capacities in renewal proliferation and hair cell regeneration. Manipulation of c-MYC and FGF pathways could be explored for mammalian hair cell regeneration.

## Introduction

The main cause of deafness in human is the loss or degeneration of sensory hair cells (HCs) in the inner ear. The mammalian inner ear does not spontaneously regenerate HCs after damage or cell death. In contrast, in birds and fish, HCs can be regenerated following HC death, leading to hearing restoration [1–4]. HC regeneration in the non-mammalian vertebrates is achieved by proliferation of supporting cells (SCs) that subsequently differentiate into new HCs. Adult mammalian SCs lack the capacity to divide or transdifferentiate, thus hearing loss as the result of HC loss is permanent. Identification and characterization of key regeneration pathways in chick and fish will likely provide insight into the regeneration process with the tools that can be tested for similar HC regeneration in mammals.

Despite the work in non-mammalian vertebrates over the years, the essential pathways that govern HC regeneration are still largely unknown. To establish a model by which key HC regeneration pathways can be identified and studied, we used microarray to profile gene expression during HC regeneration in the chick basilar papilla (BP). We subsequently used the zebrafish lateral line HC regeneration model to study the functional significance of the candidate pathways. Like HCs in chick BP, the HCs in zebrafish lateral line neuromasts can be regenerated from SCs after HC loss by ototoxic drugs [5–8]. The HCs in the neuromasts are structurally and functionally similar to mammalian HCs. Further due to their localization on the surface of the body, they are accessible to various treatments to induce hair cell death and regeneration, and can be visualized in live fish.

We report here that microarray analysis of chick BP identified two pathways, c-MYC and FGF, that are activated during HC regeneration. By specific inhibition of each pathway, we show that both are essential in HC regeneration in zebrafish lateral line neuromasts, with the primary roles in proliferation and differentiation, respectively. We further show that *fgfr* expression likely defines the organization of neuromast SCs, with *fgfr1a*-positive SCs serving as HC precursors; whereas *fgfr1a*-negative SCs serving as more primitive progenitor cells.

## Results

### Microarray analysis of HC regeneration in a chick model

In non-mammalian vertebrates such as chick and fish, HCs are readily regenerated after HC loss [3, 9] mainly from SC proliferation, and to a lesser degree from SC to HC transdifferentiation [1, 2, 4]. In gentamicin treated chick BP, most damaged HCs are ejected from the sensory epithelium by 48 hours (hrs) after injection. New HCs are regenerated first from transdifferentiation and later on from SC proliferation. The DNA synthesis peaks at 72 hrs after gentamicin injection [1, 10]. As the genes and pathways differentially expressed at these two time points should be critical for chick HC regeneration, we performed microarray analysis on the chick BP at 48 and 72 hrs after gentamicin.

By comparison of the basal region of the BP of chicks treated or untreated with gentamicin, microarray identified 1097 and 987 genes to be up- or down-regulated by more than 1.5-fold at 48 hours (hrs); whereas 1649 and 1010 genes to be up- or down-regulated at 72 hrs. Between the two time points, 500 and 435 genes were commonly up- or down-regulated (S1A Fig and S1 Table).

We performed DAVID (the Database for Annotation, Visualization and Integrated Discovery) to identify the enriched Gene Ontology (GO) pathways. The top enriched pathways upregulated in biological process included microtubule cytoskeleton (2.2%), DNA metabolic process (2.8%), cell cycle (2.7%), and ATP binding (7.6%); whereas the enriched downregulated pathways included extracellular matrix (3.4%), biological adhesion (3.7%), ear development (1.0%) and phosphorylation (4.3%) (S2 Table). Broadly, cell cycle pathways were significantly activated whereas differentiation pathways were significantly downregulated during chick HC regeneration.

Additionally, we performed the Ingenuity Pathway Analysis (IPA) to identify interactive networks. The IPA showed the top enrichment in the interactive networks involving cell cycle, cell cycle control and chromosomal segregation, hereditary breast cancer signaling and mismatch repair in eukaryotes. Thus by both the GO classification and IPA network analysis we identified enrichment in cell cycle expression, strongly supporting that proliferation is the hallmark of chick HC regeneration (S1B Fig).

To confirm the general conclusion of the microarray analysis, we performed semi-quantitative RT-PCR and *in situ* hybridization for selected genes (S2C–S2E Fig). Our analysis showed an overall agreement between microarray and RT-PCR/*in situ*, which provides the basis for the subsequent studies of the selected genes using the zebrafish model.

### Myc and Fgf in zebrafish HC regeneration

Among the genes and pathways differentially regulated during chick HC regeneration, *c-MYC* and *FGFs* drew particular interest: *c-MYC* (*MYC*) was identified as a central node in the merged top three networks (S2A Fig); whereas FGF pathway was prominently affected by differential expression of multiple member genes (S2B Fig). c-MYC is known for its role in initiation of proliferation and FGF in the development of inner ear [11–14].

**Fig 1:**
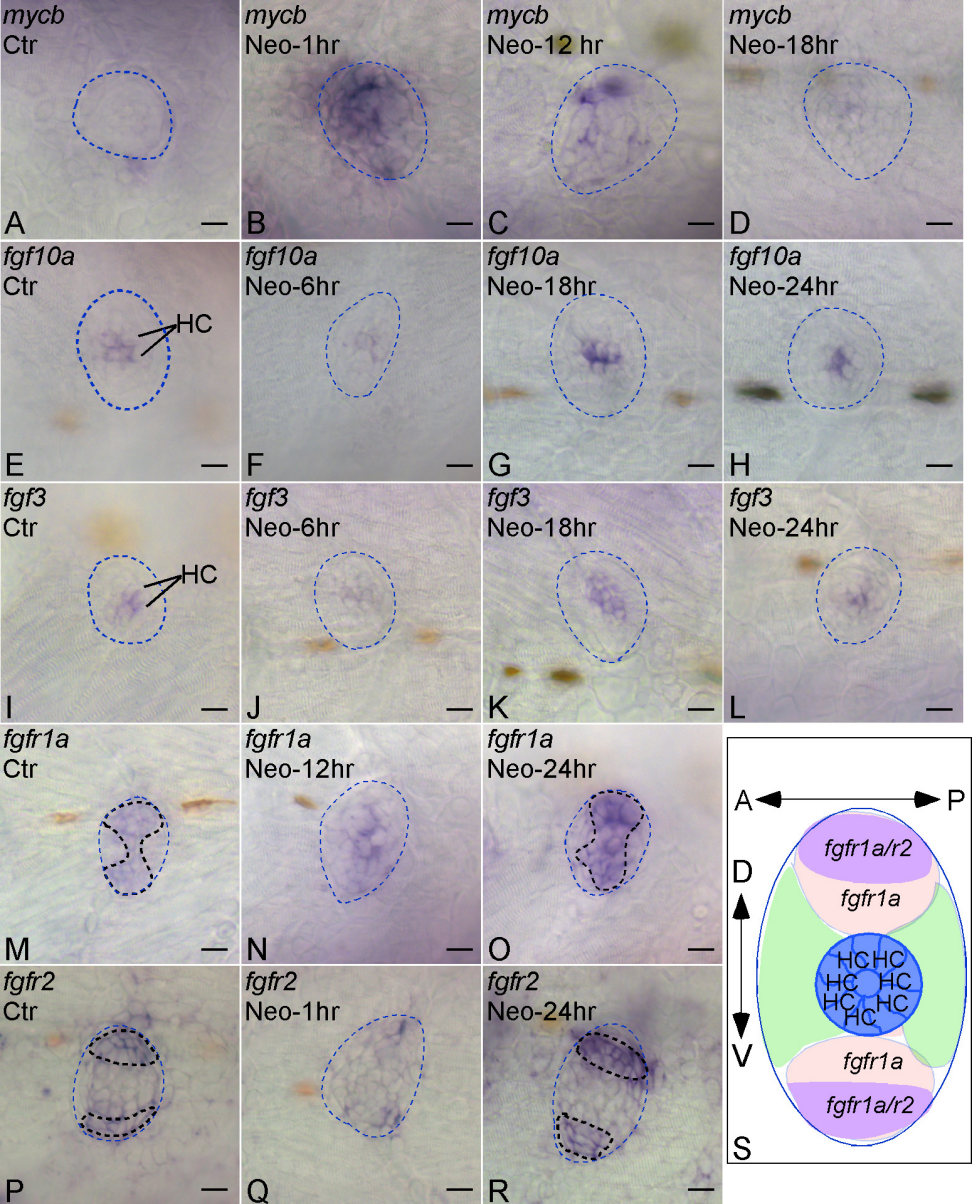
Expression of Myc and Fgf pathway genes during HC regeneration in zebrafish neuromasts by *in situ* hybridization. *mycb* (A-D), *fgf10a* (E-H), *fgf3* (I-L), *fgfr1a* (M-O), *fgfr2* (P-R) expression were shown in the lateral line neuromast L1 from 5-dpf zebrafish at different time points following neomycin treatment (e.g. Neomycin-6hr, 6 hrs after neomycin treatment). Ctr, untreated fish larvae. Dotted blue lines marked the boundary of neuromasts. Bold dotted lines marked the expression areas (M,O,P,R). (S) An illustration of a neuromast demarcated by differential *fgfr* expression patterns, including HC region, *fgfr1a(+)* only region, *fgfr1a/r2(+)* region and *fgfr1a(-)* region. A-P, anterior-posterior; D-V, dorsal-ventral. Scale bars: 10 μm.

To study the functional significance of c-MYC and FGF, we performed *in situ* hybridization to examine expression of *c-myc* and *fgf*/*fgfr*s (*fgf3*, *10a* and *fgfr1a*, *r2*) during zebrafish neuromast HC regeneration after neomycin treatment. We chose to use neomycin-induced HC death model for HC regeneration study as it is a well-established model that served to uncover the underlying regeneration mechanisms previously [5, 15–17]. We confirmed that after neomycin treatment most HCs were killed (0.7 ± 0.2 remaining HC per neuromast, mean ± SEM; n = 11). There are two orthologs of *c-Myc* in zebrafish: *myca* and *mycb* [18]. Only *mycb* was prominently up-regulated in most SCs within the boundary of mantle cells immediately after neomycin treatment, with the up-regulated expression that lasted for 12 hrs before it returned to pre-treatment base level by 18 hrs (Fig 1A–1D). Up-regulation of *c-Myc* in chick and zebrafish HC regeneration supports a conserved role in HC regeneration.

For *fgf* family members, we found generally low expression of *fgf3*, *10a* and *fgfr1a* and *r2* in the untreated control 5-day-post-fertilization (dpf) neuromasts, with *fgf3* and *10a* restricted to HCs and *fgfr1a* and *r2* restricted to SCs (Fig 1E–1R). Upon neomycin treatment, *fgf3* and *10a* expression disappeared, coinciding with HC loss, then up-regulated by 18 hrs in the center of neuromasts, in the cells that were likely to become future HCs. *fgf3* and *10a* expression returned to the pre-treatment level by 24 hrs (Fig 1E–1L). In the untreated neuromasts, *fgfr1a* was distributed along the dorsal-ventral pattern whereas *fgfr2* was distributed in the extremities of dorsal and ventral poles (Fig 1M and 1P). 12 hrs after neomycin treatment, *fgfr1a* was mainly in the center of the neuromasts with down-regulation of *fgfr2*. By 24 hrs both genes were re-established at higher levels with patterns similar to the pretreatment (Fig 1M–1O and 1P–1R).

### Myc is required for neuromast HC regeneration

Given the prominent role of Myc in cell cycle in general and its up-regulation in chick and zebrafish HC regeneration, we hypothesized that Myc is required for HC regeneration by promoting proliferation.

**Fig 2:**
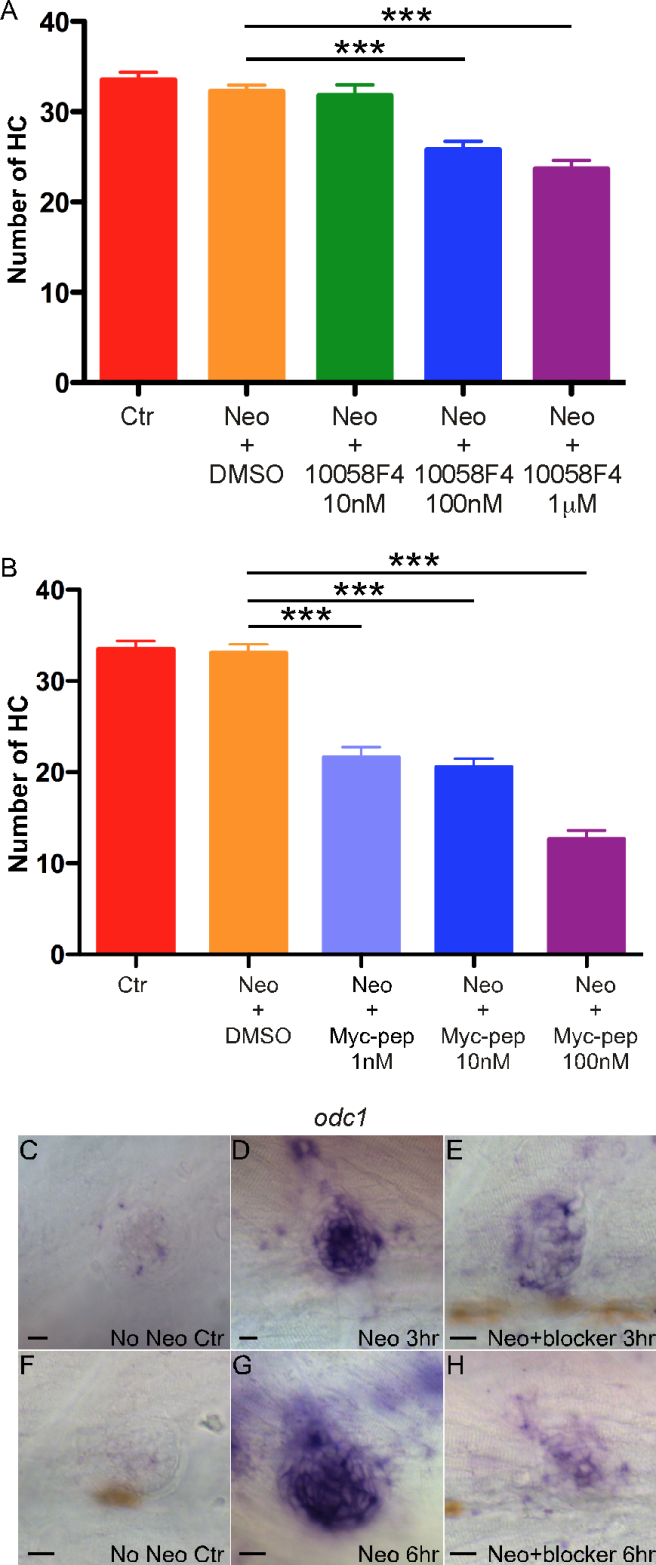
Myc inhibition suppresses HC regeneration in zebrafish neuromasts. (A) HCS1-labeled HC number was significantly reduced after treatment by c-MYC inhibitor 10058F4 that was dose-dependent. ***p<0.*001*. n = 20 larvae for each group. (B) A significant reduction in HC number was observed after the treatment by Int-H1-S6A, F8A, a peptide inhibitor of c-MYC, which was dose-dependent. ***p<0.*001*. n = 15 larvae for each group. Three independent experiments were performed for each comparison study with similar results. (C-H) Inhibitor Int-H1-S6A, F8A blocked the expression of *c-myc* target gene *odc1*. *In situ* hybridization showed up-regulation of *odc1* expression in the neuromast 3 or 6 hrs after neomycin treatment (D, G); whereas Int-H1-S6A, F8A markedly suppressed *odc1* expression (E, H). Scale bars: 10 μm.

To study the hypothesis, we blocked Myc by two independent inhibitors after neomycin-induced HC death and evaluated the effects on HC regeneration 72 hrs later. With a MYC chemical inhibitor 10058F4, a cell-permeable thiazolidinone compound that specifically inhibits c-MYC-MAX interaction and prevents the transactivation of *Myc*-targeted gene expression [19], we found a significant reduction in the number of HCs regenerated in the neuromasts compared with the DMSO-treated or neomycin-only treated controls (S3 Fig and Fig 2A). The reduction in HCs regenerated is dose-dependent (Fig 2A). At the highest dose used without causing developmental abnormalities or fish death (1 μM), the reduction was 27%. Treatment with 10058F4 in controls did not affect the HC number significantly.

We further studied the effect of a cell-permeable MYC-specific peptide inhibitor, Int-H1-S6A, F8A [20]. 72 hrs after Int-H1-S6A, F8A treatment, more dramatic dose-dependent reduction in HC regeneration was observed with over 60% reduction at the highest concentration (100 nM, Fig 2B). To confirm the specificity of Myc inhibition, we studied expression of *Odc1*, a *c-Myc* direct target in both mammals and fish [21, 22], by *in situ* hybridization. At 5 dpf, *odc1* was weakly expressed in the neuromast, then rapidly up-regulated in the neuromast SCs after neomycin treatment, coinciding with the up-regulation of *myc* (Fig 2C, 2D, 2F and 2G). Three hrs after Int-H1-S6A, F8A treatment, *odc1* was greatly reduced, whereas by 6 hrs the expression of *odc1* was negligible, in contrast to prominent *odc1* expression in the control neuromast treated with neomycin alone (Fig 2D, 2E, 2G and 2H). Blockade of *odc1* expression by Int-H1-S6A, F8A again demonstrated the specificity of Myc inhibition.

To evaluate if the Int-H1-S6A, F8A effect on HC regeneration is reversible, Int-H1-S6A, F8A was added to the media of neomycin-treated fish larvae for three days followed by the replacement with fresh media for additional three days. HCS1 labeling and counting revealed that the number of HCs regenerated after Int-H1-S6A, F8A removal was fully recovered to the normal level, demonstrating the inhibition is reversible (S4 Fig).

### Myc is required for proliferation during HC regeneration

**Fig 3:**
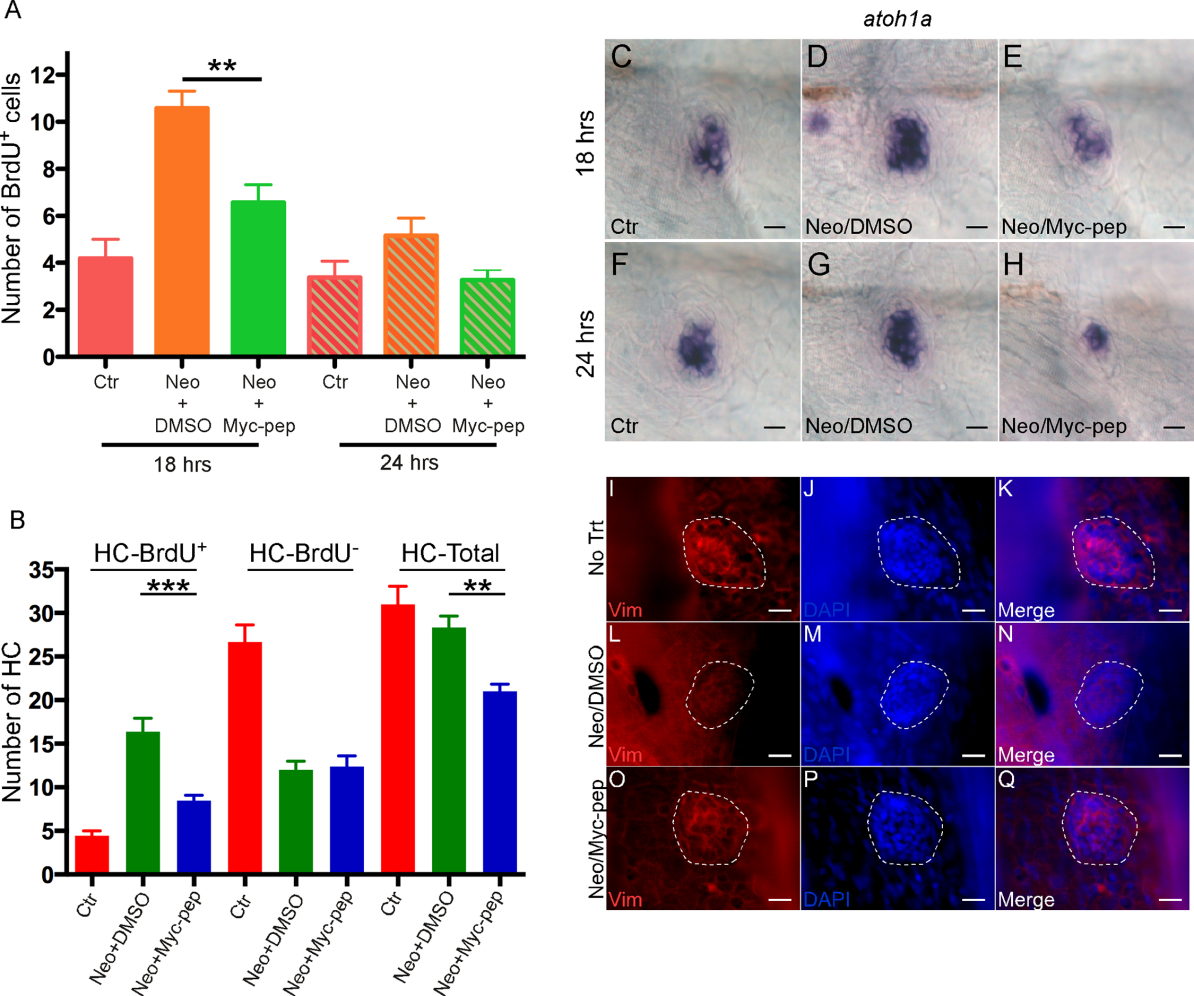
Inhibition of c-Myc blocked proliferation and down-regulated vimentin during HC regeneration. (A) A significant reduction in the number of proliferating neuromast cells (BrdU^+^) was seen 18 or 24 hrs after c-MYC inhibitor Int-H1-S6A, F8A (Myc-pep, 100 nM) treatment. Ctr, time-matched control fish without neomycin treatment. (B) c-MYC peptide inhibitor mainly blocked proliferation-derived HCs (HC-BrdU^+^) at 72 hrs. ***p<0.001; **p<0.*01*. A and B, n = 15 for each group. Two independent experiments were performed with similar results. (C-H) *In situ* hybridization of *atoh1a* on 5-dpf zebrafish neuromasts 18 (C-E) or 24 hrs (F-H) after neomycin treatment, with either Myc-pep or DMSO in the media. Ctr, time-matched control fish without neomycin treatment. *atoh1a* up-regulation by neomycin treatment was blocked in the Myc-pep treatment group (D-E, G-H). (I-Q) Vimentin was down-regulated by c-Myc during HC regeneration. 5-dpf neuromasts were labeled with vimentin and DAPI 18 hrs after neomycin treatment, with either Myc-pep (O-Q) or DMSO (L-N) in the media. No Trt, time-matched control fish without neomycin treatment (I-K). Scale bars: 10 μm.

Rapid up-regulation of *myc* after HC death is consistent with its role as an immediate-early gene involved in initiation of cell cycle [11, 14]. To determine if *myc* is involved in proliferation, Int-H1-S6A, F8A was added to the media after neomycin treatment, with BrdU added at 18^th^ and 24^th^ hr for 1 hr, respectively. 72 hrs after neomycin treatment, the number of BrdU-positive cells in the neuromasts was significantly reduced in the Int-H1-S6A, F8A-treated fish larvae compared to controls (Fig 3A), demonstrating the reduction in proliferating cells. The reduction was more prominent at 18 hrs than 24 hrs, consistent with the peak timing of cell cycle re-entry at 18 hrs [5]. To further study if reduced proliferation is responsible for reduction in the regenerated HCs, we added BrdU to the media immediately after neomycin treatment for 72 hrs, to label precursors that divide and transdifferentiate into HCs. Quantification showed significantly reduced BrdU^+^ HCs in the c-MYC peptide inhibitor group compared to the DMSO group, but no significant difference in the number of BrdU^-^ HCs between the two groups (Fig 3B). Thus c-Myc inhibition primarily blocks regeneration of HCs derived from proliferating precursors.

To determine if Myc blockade induced HC reduction is due to the toxicity of the reagent, we used TUNEL assay to study the effect of Int-H1-S6A, F8A on cell death, as the inhibitor was shown to induce apoptosis *in vitro* [20]. We found no significant difference in the number of apoptotic cells between inhibitor-treated and untreated samples following HC regeneration (S5 Fig). Our results showed that the highest concentration of c-MYC peptide inhibitor we used does not induce apoptosis in the neuromasts, and the reduction in HCs regenerated is the outcome of the attenuated proliferation of the progenitor cells.

Following HC death, *atoh1* is rapidly up-regulated in the HC precursors that divide to produce new HCs [23]. Blockade of c-Myc that inhibits proliferation could reduce the *atoh1* precursors. By *in situ* hybridization we found that the number of *atoh1a*^*+*^ cells and the level of *atoh1a* expression were significantly reduced in the Int-H1-S6A, F8A- treated fish larvae, compared to neomycin-treated controls (Fig 3C–3H). Thus *c-myc* is likely to be directly responsible for proliferation of HC precursors generated after HC death.

c-Myc is known to play a role in down-regulation of genes in wound healing and regeneration [24, 25]. We examined a filament gene vimentin after Int-H1-S6A, F8A inhibition (Fig 3I–3Q). Vimentin was significantly down-regulated following HC death by neomycin (Fig 3I and 3L), whereas it was largely maintained upon Int-H1-S6A, F8A treatment (Fig 3I and 3O), suggesting it is likely a target for c-Myc and may be involved in HC regeneration in zebrafish.

### Fgf is required for neuromast HC regeneration

FGF signaling is known to be involved in early inner ear development including HCs [12, 13]. Dynamic expression change of the FGF members during HC regeneration strongly indicates their involvement in the process. We studied the functions of Fgf in HC regeneration by two methods: by blocking Fgf pathway with a small molecule FGFR-specific inhibitor SU5402 that inhibits the tyrosine kinase activity of FGFR1-4 by interacting with the catalytic domain [26], and with genetic blockade of Fgf signaling using a transgenic zebrafish model.

**Fig 4:**
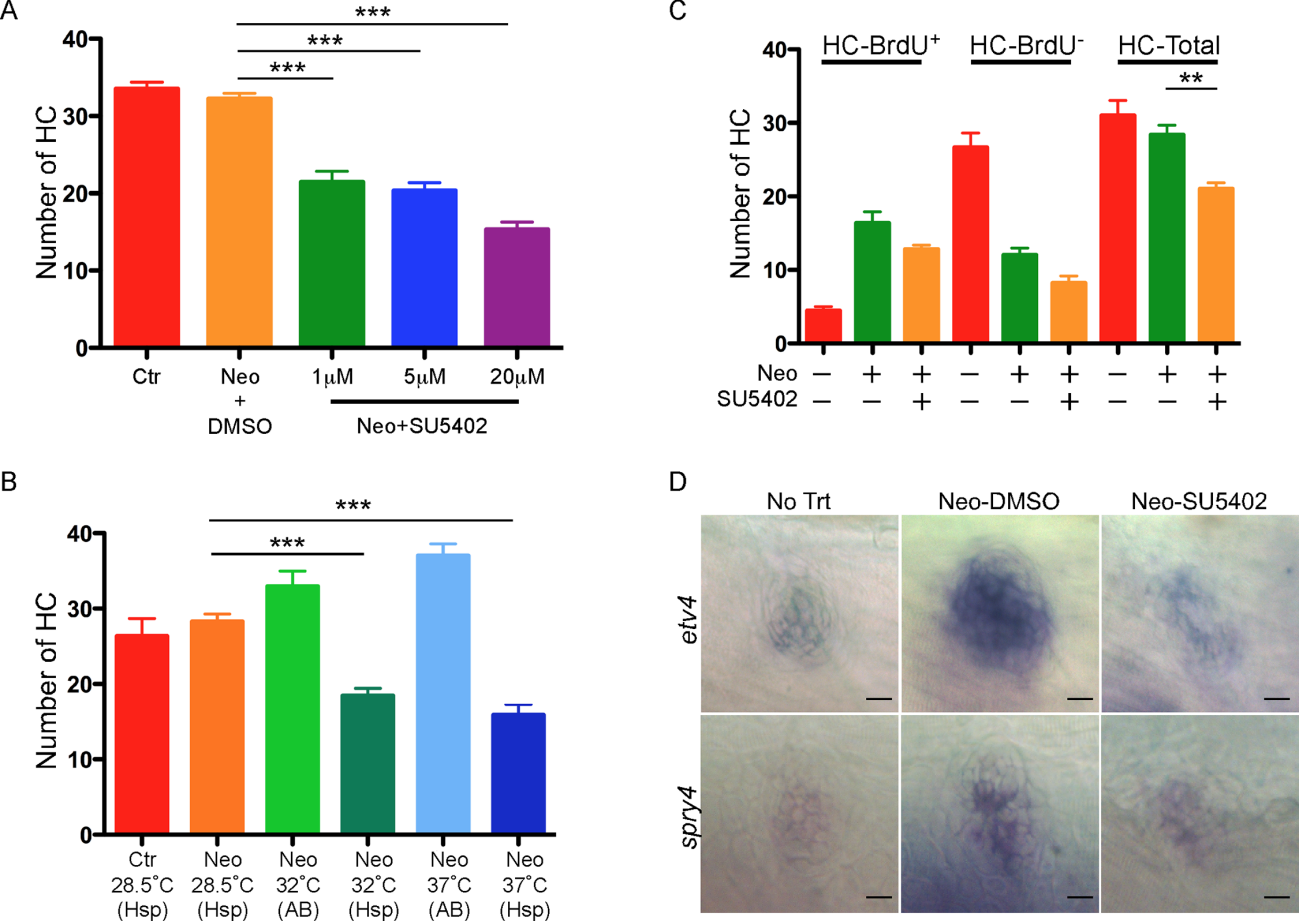
Inhibition of Fgf signaling suppresses HC regeneration. (A) A significant reduction in the number of HCs (HCS1^+^) regenerated after SU5402 treatment that was dose-dependent. Ctr, time-matched fish without neomycin treatment. (B) A significant reduction in the number of HCs regenerated in *hsp70l*:*dn-fgfr1*:*GFP* (Hsp) zebrafish neuromasts at 32°C or 37°C. AB: wild type AB fish. Ctr, time-matched transgenic fish without neomycin treatment. (C) A significant reduction in proliferation and transdifferentiation derived HCs after 20 μM SU5402 treatment for 72 hrs. ***p<0.001; **p<0.*01*. For all statistical analysis, n = 15 for each group. Three (for A) and two (for B,C) independent experiments were performed with similar results. (D) Fgf targets *etv4* and *spry4* were down-regulated by SU5402 (20 μM) in L1 neuromasts during HC regeneration. Scale bars: 10 μm.

After neomycin treatment, 5-dpf fish larvae were treated by SU5402 of varying concentrations for 72 hrs, with DMSO treatment as control. Quantification and comparison of HCS1^+^ HCs in the SU5402-treated and control groups showed a significant reduction in the number of HCs regenerated after Fgfr blockade that was dose-dependent (S3 Fig and Fig 4A). At the highest concentration used in the following studies (20 μM) the reduction was ~37% (Fig 4A).

To further demonstrate the requirement of the Fgf pathway in zebrafish HC regeneration, we studied a transgenic zebrafish line with the heat-inducible, dominant-negative *fgfr1a* tagged with GFP (*hsp70l*:*dn-fgfr1*:*GFP*)[27]. In this line, Fgfr signaling is blocked when zebrafish are subject to heat shock. After neomycin treatment, the *hsp70l*:*dn-fgfr1*:*GFP* zebrafish were either placed at 28.5°C (control), 32°C or 37°C for 3 hrs daily for 3 days. Heat shock led to activation of dn-*fgfr1a*, which is predicted to block all Fgfr-mediated signaling [27]. *In situ* hybridization showed down-regulation of the Fgf downstream genes *etv4* and *spry4* (S6 Fig). After heat shock (32°C or 37°C), a significant suppression of HC regeneration was observed, compared to the zebrafish kept at 28.5°C. The degree of suppression corresponded to the temperatures: there was more dramatic suppression (48%) at 37° on HC regeneration than at 32°C (35%) (Fig 4B). Heat shock alone, at 32°C or 37°C, did not reduce but increased the number of HCs regenerated in the non-transgenic AB fish larvae (Fig 4B). Although heat shock is known to protect HCs from apoptosis [28], its role in HC regeneration is unknown. Thus genetic blockade of Fgfr signaling led to significant reduction in neuromast HC regeneration. Combined with the results from SU5402, we conclude that Fgf signaling is required for neuromast HC regeneration.

To study SU5402 inhibition on the Fgf pathway genes, we checked expression of two Fgf target genes: *etv4* (also named *pea3*) [29, 30] and *spry4* [31]. *In situ* hybridization showed that the expression of both was up-regulated 24 hrs after neomycin treatment, and the up-regulation was suppressed in the fish treated with SU5402 for 24 hrs (Fig 4D). Thus suppression of Fgf signaling by SU5402 led to significant and specific down-regulation of Fgf target genes.

Both small molecule inhibitors of c-Myc and Fgf suppressed neuromast HC regeneration but have no effect on normal HCs (S7 Fig). As another control, we tested inhibition of the TGF-β1 pathway that did not show significant change in chick HC regeneration. Suppression of Tgf-β1 by an inhibitor ([3-(pyridin-2-yl)-4-(4-quinonyl]-1H-pyrazole) [32] over a range of concentrations did not significantly affect the number of HCs regenerated after neomycin treatment (S8 Fig), further supporting the pathway-specific inhibition of c-Myc and Fgf.

### Fgf is involved in HC regeneration through proliferation and transdifferentiation

Activation of Fgf signaling is involved in regeneration in zebrafish tissues such as fins by promoting proliferation [27]. As proliferation is a major mechanism underlying neuromast HC regeneration in zebrafish [5–7, 33], we studied the effect of Fgf blockade on proliferation during HC regeneration by BrdU labeling.

After neomycin treatment, BrdU and SU5402 were added to the media for 72 hrs. Interestingly while there was a reduction in both BrdU^+^ and BrdU^-^ HCs, none of which was significant compared to control. Combined, however, there was a significant reduction in the number of HCs after SU5402 treatment (Fig 4C). The data shows that Fgf blockade inhibits HC regeneration, possibly by attenuating both proliferation and transdifferentiation in neuromast HC regeneration.

### *fgfr1a* demarcates HC precursors with different potential from progenitors

At 5 pdf, *fgfr1a* is distributed along the dorsal-ventral axis of the lateral line neuromasts (Fig 1M–1O), a region in which HC precursors divide to produce new HCs after HC death [33]. *fgfr2* is restricted to a 2–3 cell layer region along the dorsal-ventral axis (Fig 1P–1R). Based on the expression pattern of *fgfr1a*, a lateral line neuromast can be subdivided into five distinct regions: the central region occupied by HCs, the dorsal and ventral *fgfr1a*(+) regions, and the posterior and anterior *fgfr1a*(-) regions (Fig 1S). Given the role of *fgf* in neuromast HC regeneration and the distribution of *fgfr1a*, we hypothesized that *fgfr1a* marks the dorsal-ventral HC precursors with the capacity to directly regenerate HCs; whereas the anterior-posterior *fgfr1a*-negative (*fgfr1a*(-)) cells are more primitive progenitors with potential to replenish HC precursors.

To study how different regions contribute to HC regeneration, we used a Ti:Saphire laser to ablate the SCs based on the *fgfr1 in situ* pattern within the L1 neuromast, in conjunction with HC ablation. We used compound transgenic fish larvae for *pou4f3*:*GFP* (labeling HCs) [34] and *ET20* (labeling mantle cells) [35], in which both HCs and some SCs are GFP-positive (S9A Fig), for laser ablation. Three types of ablation were performed: HCs only (S9A and S9B Fig); HC/*fgfr1a*(+) SCs (S9C and S9D Fig), and HC/*fgfr1a*(-) SCs (S9E and S9F Fig). For SC ablation, we ablated 10 cells in each dorsal and ventral *fgfr1a*(+) region (S9C and S9D Fig), or anterior and posterior *fgfr1a*(-) region (S9E and S9F Fig), adjacent to HCs, respectively. *In situ* hybridization confirmed the ablation of *fgfr1a*(+) SCs: *fgfr1a* was absent only in the neuromasts with *fgfr1a*(+) ablation (S9G Fig). The laser ablation is approximate, as we could not ablate all *fgfr1a*(+) cells accurately due to the lack of *fgfr1a* fluorescence signal. Nevertheless our approach did allow us to cover a majority of *fgfr1a*(+) or *fgfr1a*(-) cells judging by *in situ* hybridization after the ablation.

**Fig 5:**
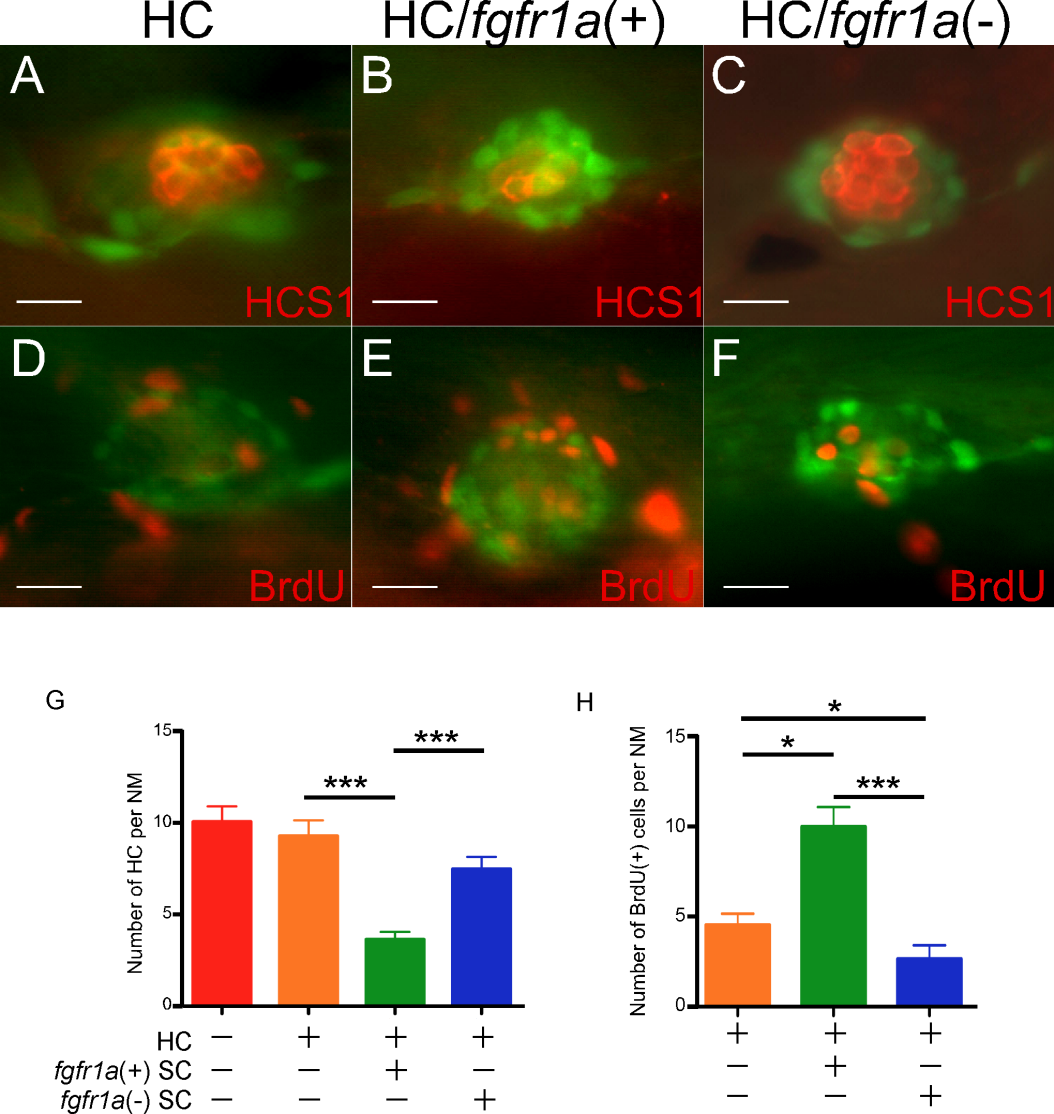
Neuromast SCs with different capacities in HC regeneration and proliferation. Hybrid larvae of *pou4f3*:*GFP* and *ET20* fish ablated in HCs only (A,D), HC/*fgfr1a*(+) SCs (B,E), or HC/*fgfr1a*(-) SCs (C,F) were stained with HCS1 (A-C) or BrdU (D-F) antibody, to illustrate proliferating (BrdU^+^) and regenerated HCs (HCS1^+^). (G) Quantification showed that only HC/*fgfr1a*(+) SC ablation significantly reduced the regenerated HCs. (H) Quantification showed that HC/*fgfr1a*(+) SC ablation increased the number of proliferating cells; whereas HC/*fgfr1a*(-) SC ablation reduced the proliferating cells. (G,H) ***p<0.001; *p<0.*05*. n = 12 in each group. Scale bars: 10 μm.

Following laser ablation, fish larvae were returned to media with BrdU added for a total of 72 hrs. We performed immunolabeling for HCs (HCS1) and BrdU, and quantified the number of HCs regenerated. If *fgfr1a*(+) SCs were HC precursors, their ablation would affect the number of HCs regenerated. Indeed the ablation of HC/*fgfr1a(+)* SCs led to a drastic reduction in the number of HCs regenerated by as much as ~60% (Fig 5B and 5G). In contrast, the ablation of HC/*fgfr1a*(-) SCs resulted in the number of regenerated HCs that was similar to the control with HC ablation alone (Fig 5A and 5C and 5G). Thus the dorsal-ventral neuromast *fgfr1a*(+) SCs, not the posterior–anterior *fgfr1a*(-) cells, contain the likely HC precursors that contribute directly to HC regeneration.

After HC precursors become HCs, the lost precursors need to be replenished. As the *fgfr1a*(-) SCs do not contribute significantly to regenerated HCs, they could have a higher potential to divide and replenish the lost HC precursors. We found a significant increase in the number of BrdU labeled cells within the neuromast after HC/*fgfr1a*(+) SC ablation, in comparison to HC ablation alone (Fig 5E and 5H). In contrast, the HC/*fgfr1a*(-) SC ablation led to a significant reduction of the BrdU^+^ cells (Fig 5F and 5H). Thus the loss of the *fgfr1a*(+) SCs induces a significant increase in proliferating SCs that are likely originated from the *fgfr1a*(-) SCs, whose loss leads to a significant reduction in proliferating cells. The results strongly support that *fgfr1a*(-) SCs have heightened capacity to divide and most likely to replace the lost *fgfr1a*(+) SCs.

## Discussion

By comparative study of gene expression profiling in the quiescent and HC-regenerating BP in chick, we identified the pathways that change significantly in the process. We focused on the early stages of regeneration (48 and 72 hrs after gentamicin), as we were particularly interested in the initiation of proliferation and early transdifferentiation, whereas hair cell differentiation markers only appear at 78 hrs in the transdifferentiated HCs and even later in mitotically regenerated HCs [1, 10]. By studying c-Myc and Fgf signaling in the zebrafish model, we provide evidence that Myc and Fgf signaling are essential in HC regeneration in the lateral line neuromasts. The study provides strong evidence that *c-Myc* and *Fgf* are likely conserved between chick and zebrafish during HC regeneration, which provides the rationale for manipulating them for HC regeneration in mammalian inner ear.

*c-Myc* is a critical gene in proliferation and growth of cells, regulates up to 15% of all genes of the genome [36, 37]. It is one of the key factors in reprogramming fibroblast cells into iPS cells [38]. *Myc* is up-regulated in chick BP and zebrafish HC regeneration [39, 40], but its role is unknown. Despite its moderate level of up-regulation (~1.6 fold) in our microarray analysis, the IPA analysis identified *c-MYC* as a central node in a highly connected network involving a large number of up-regulated proliferation genes (S2A Fig). Our zebrafish study provides compelling evidence that Myc is required for cell cycle re-entry, probably by reprogramming and propelling progenitors to proliferate. There are two zebrafish *c-Myc* orthologs, *myca* and *mycb* [18], and both are functionally similar to the mammalian *c-Myc* [41]. In the zebrafish retina only *mycb* is up-regulated during regeneration [42], and in our studies only *mycb* is up-regulated in the neuromasts immediately after neomycin treatment. Interestingly in the two recent studies on genes differentially expressed during HC regeneration after neomycin or CuSO_4_ treatment [43, 44], up-regulation of *mycb* and *odc1* was detected in one of them but not the other, which could reflect the difference in the transgenic fish lines used, the methods to kill hair cells, or the method for expression profiling. We also showed that expression of vimentin, a mesenchymal marker, was down-regulated following HC death whereas its expression was maintained by a c-Myc inhibitor. *Myc* overexpression that led to vimentin down-regulation has been reported in fibroblasts undergoing the mesenchymal-to-epithelial transition (MET) [45]. Interestingly MET is also involved in chick HC regeneration *in vitro* [46]. Combined it is suggestive of a link between Myc overexpression, vimentin down-regulation and possible MET in the context of HC regeneration.

In mammals *c-Myc* is not expressed in the developing inner ear, in which conditional knockout of *c-Myc* does not produce any phenotype [47, 48]. Among the mammalian *Myc* members (*c-Myc*, *N-Myc* and *L-Myc*), *N-Myc* is critical for inner ear morphogenesis [47, 48]. Overexpression of *c-Myc* enhances self-renewal in otic progenitor cells while maintaining their differentiation capability [49]. In adult mouse utricle, overexpression of *c-Myc* induces proliferation of SCs *in vitro* [50]. Thus activating *c-Myc* in the mammalian inner ear could have a similar effect as in zebrafish, to promote proliferation and HC regeneration.

Multiple FGF ligands and receptors are involved in the inner ear development in mice [12, 13]. The knockout/overexpression of *Fgf3*, *8*, *10* cause various defects in otocyst development [51–55]. Knockout of *Fgfr1* resulted in severe HC loss only in cochlea [56]; whereas *Fgfr2 IIIb* knockout led to the missing sensory epithelia and endolymphatic duct [57]. It is suggested that the diverse phenotypes by FGF signaling are due to different ligand-receptor specificities [58, 59]. For instance, *Fgf3* and *10* may bind to both *Fgfr1* and *Fgfr2* with overlapping functions in the inner ear development [57]; whereas *Fgf8* induces the pillar cell fate mediated by *Fgfr3* [53]. The non-overlapping distribution of FGF ligands and receptors in mouse inner ear suggests they interact through paracrine pathway. In zebrafish, Fgf pathway genes are involved in the development of neuromasts as well as the regeneration of fins and hearts [12, 60, 61]. Expression of *fgf3 and 10a* in the HCs, and *fgfr1a and r2* in the SCs is consistent with the non-overlapping expression patterns in mice [12, 53, 56, 62]. Recent studies using FACS sorted cells found most of the Fgf pathway genes we examined were enriched in mantle cells [43, 44]. It is likely that *fgf3 and 10a* are expressed in both HCs and SCs whereas the purified cells only represent a portion of Fgf-expressing cells.

Our study supports that Fgf signaling is important in neuromast HC regeneration, which is consistent with the report showing that SU5402 blocks HC development in zebrafish inner ear [23]. Interestingly, studies on chick BP development *in vitro* showed SU5402 increased HCs, likely through SC transdifferentiation without proliferation [63]. *FGFR3* was also found to be down-regulated during hair cell regeneration in chick BP [64]. The discrepancy between those studies including ours may be due to the differences in Fgf pathways/genes examined in different species, *in vivo* vs. *in vitro*, and in regeneration vs. development. Studies by Jiang et al [43] showed that *fgf3* and *etv4* were down-regulated in zebrafish neuromasts immediately (1–3 hrs) after Neomycin treatment and returned to normal expression level at 10 hrs, which is consistent with our data (Fig 1I–1L). In a separate study, Steiner et al showed *fgfr1a* mainly in the mantle cells [44]. The discrepancy could be due to *in situ* probes used, different neuromasts examined or unknown factors, which could be resolved by future studies. Combined with our results, it is likely that Fgf pathway is initially down-regulated but later on up-regulated in the hair cell regeneration process, which is consistent with our microarray data in chick BP.

In the heat shock experiments we performed to block Fgfr-mediated signaling, we surprisingly observed suppression of HC regeneration in both 32°C- or 37°C-treated groups. The reason is not clear as *hsp70l* promoter is generally induced at 37–40°C. However, previous studies showed *hsp70l* can be moderately induced at 34°C and highly up-regulated at 37°C, compared to 28.5°C control [65]. Therefore, it is possible Fgfr signaling is partially blocked at 32°C which suppresses HC regeneration, with the suppression less potent than at 37°C. It is interesting that heat shock alone stimulated HC regeneration in wild type fish (Fig 4B), suggesting a new mechanism that can be further explored.

Our study could not address which Fgf member contributes specifically to the regeneration, and future experiments targeting specific member should reveal individual roles. Compared to Myc inhibition, after Fgf blockade, proliferation and direct transdifferentiation were both attenuated but not significantly by statistics analysis (Fig 4C). However, the combinatory effect of both leads to a significant reduction in the HCs regenerated, an indication of more subtle and multi-functional role of Fgf, in contrast to the prominent and singular role of Myc in proliferation.

It is of great interest to identify the cell subtypes with different potentials in HC regeneration in zebrafish. Previous studies using real-time recording elegantly demonstrated that after drug-induced HC death, HC precursors undergo symmetric division to give rise to pairs of new HCs [6, 7, 33]. Our laser ablation study provides strong evidence to support that the HC precursors are primarily located in the dorsal-ventral compartments that is marked by *fgfr1a* expression; whereas the SCs, located in the anterior-posterior compartments largely devoid of *fgfr1a*, are the progenitors with heightened potential in proliferation (Fig 5). *fgfr1a* expression overlaps with *fgfr2* at the extreme dorsal-ventral polarity regions. The *fgfr1a*^+^/*r2*^+^ SCs may be more primitive than *fgfr1a*^+^ SCs, as *Fgfr2* plays an earlier role in the developing inner ear than *Fgfr1* [57]. SCs in the anterior-posterior compartments may be more primitive progenitors by enhanced division and migration to replace the cells in the dorsal-ventral regions. Using live imaging techniques, Wibowo et al [7] showed that new HC progenitors are located at the dorsal-ventral poles of the neuromasts. They also suggested that the HC progenitors migrated from elsewhere in the neuromast to the dorsal/ventral area before regeneration. Both of the results are consistent with our findings. Interestingly, a recent study using the same technique [6] showed the dorsal/ventral area is enriched with BrdU^+^ SCs, yet neither dividing nor quiescent SCs move much during HC regeneration. They suggested that the SCs at the dorsal/ventral area divide, while the HCs are derived from quiescent SCs at the center and anterior/posterior regions. While our laser ablation study supports the hypothesis by Wibowo et al, future thorough examination will be needed to better understand the differences. We also noticed that the expression patterns of *in situ* results in Fig 1 and S9 Fig are somewhat different. One possibility is that laser ablated fish went through multiple steps during the process, which may affect the *in situ* patterns.

Previous studies [6, 7] showed *atoh1a* is enriched in the dorsal/ventral poles whereas the Notch activity is outside the polar area. Both Notch and Wnt pathways are involved in proliferation and HC regeneration. Expression patterns of several Notch and Wnt pathway genes resemble *fgfr1a* or *fgfr2*, suggesting Fgf pathway and Notch/Wnt pathway may interact with each other during HC regeneration. It will be informative to use genetically marked *fgfr1a*^+^/*r2*^+^ SCs, as well as the anterior-posterior SCs, to determine different SC subtypes within a neuromast and specific signaling pathways active in the SC subtypes. Understanding the difference of SC subtypes on the molecular level could provide insight to HC regeneration by SC in mammalian inner ear.

Our study illustrates the utility using whole genome approach to comprehensively survey gene expression, to identify and test candidate pathways using spontaneous HC regeneration model. It provides a route that other signaling pathways can be similarly studied. Our study utilized the pathway information extensively to provide critical information that enabled us to study *Myc*, which would have been missed if the selection criteria were based on the expression level change alone. Pathway utilization has been a hallmark in our previous studies [66–69]. It is highly likely the pathway information will be even more important as we aim to manipulate them for HC regeneration in mammalian inner ear due to the availability of compounds to block or activate major pathways.

## Materials and Methods

### Microarray of chick basilar papilla and data analysis

To induce HC death, 12-16-day-old chicks were treated with gentamicin as previously described [70]. The time of injection was defined as 0 hr. The age-matched, untreated samples were used as controls. Basilar papilla samples at different time points after gentamicin treatment were dissected and the basal halves, where the HC loss mainly occur, were collected in RNA Later solution (Ambion). 4–8 samples from the same time point were pooled for RNA extraction. RNeasy Mini Kit (Qiagen) was used to extract RNA and the quality of RNA was verified on Bioanalyzer (Agilent). The use of chick was approved by the Boston Children’s Hospital IACUC committee.

For microarray studies, Biotin labeled cRNA for microarray was prepared using the small sample preparation protocol II from Affymetrix. Hybridization to GeneChip® Chicken Genome Array (Affymetrix) was done at MIT BioMicroCenter. All microarray data have been deposited in NCBI Gene Expression Omnibus Database (GEO; http://www.ncbi.nlm.nih.gov/geo/) with the accession number GSE79963. The normalization of array data and calculation of expression levels were done in dChip [71]. Differentially expressed genes were defined as probe sets with log_2_ (fold change of expression levels) > 0.6 or < -0.6 compared with controls as described before [67].

Annotations of genes are based on the NetAffx Annotation Release 34 (November 2013) from Affymetrix. Analysis of Gene Ontology (GO) categories was done using DAVID 6.7 (http://david.abcc.ncifcrf.gov/)[72]. Redundant probe sets with the same Entrez Gene ID were excluded for GO analysis. Clusters with enrichment score > = 1 were picked and the top GO categories with FDR < = 10% were presented. For pathway analysis, mouse orthologs of the chick differentially expressed genes were identified and the corresponding probe sets on Affymetrix Mouse Genome Array (MOE430 2.0) were analyzed through Ingenuity Pathways Analysis (IPA, Ingenuity® Systems, www.ingenuity.com).

### Zebrafish husbandry and fish treatments

Adult zebrafish lines (*AB* and *Albino*) were purchased from ZFIN. Additional zebrafish were gifts from the following sources: *ET4* and *ET20*, Dr. V. Korzh (Institute of Molecular and Cell Biology, Singapore); *pou4f3*:*GFP*, Dr. H. Baier (UCSF, CA); *hsp70l*:*dn-fgfr1*:*GFP*, Dr. K. Poss (Duke University). Standard procedures in maintenance of zebrafish were as described by Westerfield [73]. Embryos were produced by paired mating and raised in egg water at 28.5°C. Embryos and larvae were maintained at a density of 50–60 per 100 mm^2^ dish. Zebrafish use was approved by the Massachusetts Eye & Ear Infirmary IACUC committee.

Neomycin treatments were performed on 5–6 dpf larvae because of the relative insensitivity of zebrafish to neomycin before 5 dpf [16, 17]. *AB* fish larvae were used for experiments unless specified otherwise. Free-swimming larvae were treated with 200 μM neomycin solution for 1 hr, rinsed three times, and allowed to recover in fresh egg water at 28.5°C [15–17].

### Proliferation and inhibition studies in zebrafish

For proliferation assay, BrdU (10 mM) or EdU (1 mM) were added into water at specific time points for varying periods. Sibling fish were mock-treated by transferring into neomycin-free egg water and served as controls. For inhibitor study, all compounds were dissolved in DMSO to the specified concentrations. DMSO served as untreated control. For c-Myc inhibition, after neomycin treatments, the larvae were treated with small molecule chemical inhibitors (10058F4, 5-[(4-Ethylphenyl)methylene]-2-thioxo-4-thiazolidinone, Calbiochem) or peptide inhibitors (Int-H1-S6A, F8A, Enzo Life Sciences) for different time points and doses as indicated in the text. For Fgf pathway inhibition, the larvae were treated with FGFR inhibitor SU5402 (3-[3-(2-Carboxyethyl)-4-methylpyrrol-2-methylidenyl]-2-indolinone, Calbiochem). None of the inhibitors exhibited toxicity to HCs in fish without neomycin (S7 Fig). An inhibitor of TGFBR1 (TGFBR1I, [3-(Pyridin-2-yl)-4-(4-quinonyl)]-1H-pyrazole, Calbiochem) was also used to show the inhibition is pathway-specific (see text). For heat shock experiments, embryos from *hsp70l*:*dn-fgfr1*:*GFP* transgenic fish were incubated in pre-warmed water bath at 32°C or 37°C for 1 hr per day for three days.

### HC labeling and immunohistochemistry

HCs were labeled with the vital dye, Yo-Pro-1 (Invitrogen) as described [17]. Briefly, larvae were placed in egg water containing 5 μM Yo-Pro-1 for 30 min and rinsed three times in fresh egg water. They were then anesthetized with 0.02% Tricaine (MS-222, Sigma) and the number of HCs was counted from three fixed locations of lateral line neuromasts (SO1, SO2, L1) at room temperature.

For immunohistochemistry, fish larvae were anesthetized as described above, then fixed in 4% paraformaldehyde (PFA) in PBS, pH 7.4, overnight at 4°C. After fixation, samples were washed three times with PBS-T (PBS/0.1% Triton X-100). Mouse anti-HCS-1 (HC-specific antigen; 1:100; a gift from Jeff Corwin; University of Virginia, Charlottesville, VA) and rat anti-BrdU (1:100, Sigma) were used to visualize HCs and cells undergoing cell cycle. Mouse Vimentin antibody for immunostaining was from Sigma (V5255). BrdU immunostaining was done following the protocol described before [5]. Click-IT^®^ EdU labeling kit (Invitrogen) was used to visualize the EdU uptake. For TUNEL assay, we used ApopTag^®^ Fluorescein In Situ Apoptosis Detection Kit (Millipore) following the manufacturer’s protocol.

### *In situ* hybridization

RT-PCR was performed using a cDNA mixture prepared from AB wild-type zebrafish at stage of 5 dpf. The conditions for RT-PCR were as follows: 94°C, 2 min; 94°C, 30 sec; 68°C, 1 min; 72°C, 1 min 30 sec for 35 cycles; 72°C, 10 min. RT-PCR fragments were further cloned into TOPO TA-Cloning vector (Invitrogen). Ribo-probes preparation and subsequent *in situ* hybridization follow the procedure described before with slight modification [74].

The sequences of zebrafish and chick primers for RT-PCR and in situ hybridization were shown in S3 Table.

### Statistical analysis

The total number of HCs and BrdU labeled cells were counted from the supraorbital neuromasts SO1, SO2 and posterior lateral line neuromast L1, except specified. We compared the treated and control groups by ANOVA and Tukey post hoc tests using the Prism 5.0 statistical analysis program (GraphPad), with the bars representing Mean (±SEMs).

### Laser ablation of neuromast cells

All Imaging and ablations were performed on a Zeiss LSM710 confocal/ 2-photon microscope setup. In fish lines not expressing GFP, HCs were labeled transiently with Yo-Pro-1. For ablation of neuromast cells, 5–6 dpf larvae were mounted laterally left side up in 1% low-melting agarose in egg water and 1.5x Tricaine (0.015% w/v). All ablations were performed on posterior lateral line neuromast 1 (L1, dorsal of the swim bladder with some variation in its lateral position). Individual cells were ablated by drawing a circular region of interest (5 μm diameter) around their nuclei and exposing it with a single 750 nm light pulse from the Chameleon 2-photon laser (Coherent) at 15% of its 2.7 W power (3.15 μs pixel dwell time, 200 nm x 200 nm pixel). Ablated neuromast regions were pairs of circles of 10 μm diameter each and applied horizontally (3 and 9 o’clock orientation) or vertically (6 and 12 o’clock orientation) when viewing the neuromast. Treated neuromasts were visually inspected 1 hr post ablation, and the rare residual HC was treated with an additional light pulse. All larvae were then unmounted and kept in egg water at 28.5°C until the time of sacrifice. To see the cells undergoing proliferation, fish larvae were incubated in the 10 mM BrdU/egg water for 72 hrs.

## Supporting Information

S1 Fig Genes differentially expressed during chick BP HC regeneration by microarray analysis. (A) Venn Diagram of genes up- and down-regulated in chick BP 48 or 72 hrs after gentamicin injection compared with control. (B) GO categories enriched in up- (top, dark blue bars) or down-regulated genes (bottom, light blue bars) using the IPA package. Mouse orthologs of differentially expressed genes in chick BP were used for analysis.
(TIF)

S2 Fig **Pathways regulated by *c-Myc* (A) and *Fgf* (B) by IPA analysis.** Genes up-regulated in chick BP during HC regeneration were marked in red and those down-regulated were marked in green. (A) *c-Myc* is a central node for the network formed by the up-regulated genes in proliferation. (B) A large number of *Fgf* members showed differential expression patterns during HC regeneration. (C) Semi-quantitative RT-PCR of selected genes from microarray studies showed differential expression in chick BP 48 or 72 hrs after gentamicin treatment. *GAPDH* is the control (Ctr). (D-E) *In situ* hybridization of *HBEGF* in the whole mount chick BP. *HBEGF* showed up-regulation in the sensory epithelium (marked by dotted lines) close to base (proximal end) 48 hrs after gentamicin treatment compared with control (0 hr). Scale bars: 50 μm
(TIF)

S3 Fig c-Myc and Fgf pathway inhibitors block HC regeneration. 5-dpf zebrafish larvae were treated with different inhibitors or DMSO after neomycin-induced HC death. 72 hrs later, the HCs were labeled with Yo-Pro-1. The pictures of whole fish (left) and enlarged neuromast L1 (right) showed the reduction of HC number in the inhibitor-treated neuromasts. Scale bars: left panel, 50 μm; right panel, 10 μm.
(TIF)

S4 Fig The inhibition by Myc peptide inhibitor and SU5402 is reversible. 5-dpf zebrafish larvae with neomycin treatment were treated for 72 hrs with 100 nM c-MYC inhibitor Int-H1-S6A, F8A (Myc-pep) or 20 μM SU5402, followed by replacement with fresh media for additional 72 hrs. HCs were labeled with HCS1 antibody. There was no significant difference in the number of HCs between the inhibitor-treated groups and DMSO-treated (Neo+DMSO) or no-treatment control (Ctr).
(TIF)

S5 Fig The Myc inhibitor does not induce apoptosis. 5-dpf zebrafish larvae with neomycin treatment were then treated with or without 100 nM c-MYC inhibitor Int-H1-S6A, F8A for 72 hrs (G-I, J-L). Larvae without neomycin and inhibitor treatment (No Trt) and larvae collected 1 hr after neomycin treatment were used as controls. The fish were stained with HCS1 antibody (A,D,G,J) to label HCs and TUNEL assay (B,E,H,K) to measure apoptosis. No significant difference in apoptosis signal was observed between inhibitor-treated and non-treated fish (TUNEL^+^ cells per neuromast: 0.4 ± 0.2 for No Trt, n = 14; 0.4 ± 0.1 for Neo 72hr, n = 15; 0.6 ± 0.2 for Neo/Myc-pep 72hr, n = 15). All TUNEL signals were from outside of the neuromast (I,L). In the positive control (D-F), a significant increase in the TUNEL^+^ cells were seen inside the neuromast. Scale bars: 10 μm
(TIF)

S6 Fig Blockade of Fgf signaling in heat shocked transgenic fish. *In situ* hybridization showed Fgf targets *etv4* (A,B) and *spry4* (C,D) were relatively down-regulated in *hsp70l*:*dn-fgfr1*:*GFP* (Hsp) zebrafish neuromasts at 37°C compared to control. Scale bars: 10 μm
(TIF)

S7 Fig The inhibitors do not affect hair cell survival. 5-dpf zebrafish larvae without neomycin treatment were treated for 72 hrs with different c-Myc and Fgf inhibitors at the highest concentrations used for our experiments. There was no significant difference in the number of HCs after the inhibitor treatment in comparison to DMSO-treated (DMSO) or no-treatment control (Ctr).
(TIF)

S8 Fig Blockade of Tgf-β1 pathway with an inhibitor (TGFBR1I) has no effect on zebrafish HC regeneration. Quantification of Yo-Pro-1-labeled HCs of the 5-dpf neomycin-treated zebrafish neuromasts with different concentrations of TGFBR1I for 72 hrs showed no effect on hair cell regeneration compared to the no-treatment control (Ctr).
(TIF)

S9 Fig Laser ablation of HCs and SCs in zebrafish neuromasts. Hybrid larvae of *pou4f3*:*GFP* and *ET20* fish were used to ablate HCs alone (A,B), and HC/*fgfr1a*(+) SCs (C-D), or HC/*fgfr1a*(-) SCs (E-F). Neuromasts before (A,C,E) and after ablation (B,D,F) were shown. The red circles are the target areas for ablation. A-P, anterior-posterior; D-V, dorsal-ventral. Scale bar, 10 μm. (G) *In situ* hybridization of *fgfr1a* confirmed the ablation of *fgfr1a*(+) cells in HC/*fgfr1a*(+) ablation group, where *fgfr1a* is undetectable; whereas in HCs or HC/*fgfr1a*(-) ablation group, *fgfr1a* signal is still present. Similar ablation did not change *fgfr2* signal. Ctr, unablated neuromasts labeled with *fgfr1a*. Scale bars: 10 μm.
(TIF)

S1 Table Genes differentially expressed in chick BP 48 or 72 hrs after gentamicin treatment. The listed included redundant probesets for some genes, i.e. genes that were represented multiple times by different probesets.
(XLS)

S2 Table GO categories enriched in up- or down-regulated genes in chick BP regeneration identified from microarray studies using DAVID analysis. (XLS)

S3 Table Primers used for RT-PCR and *in situ* hybridization. (XLS)
