# Supplementary figures and images for: Myc and Fgf Are Required for Zebrafish Neuromast Hair Cell Regeneration
Source: PLoS One. 2016 Jun 28;11(6):e0157768. doi: 10.1371/journal.pone.0157768 (PMC4924856; doi:10.1371/journal.pone.0157768)

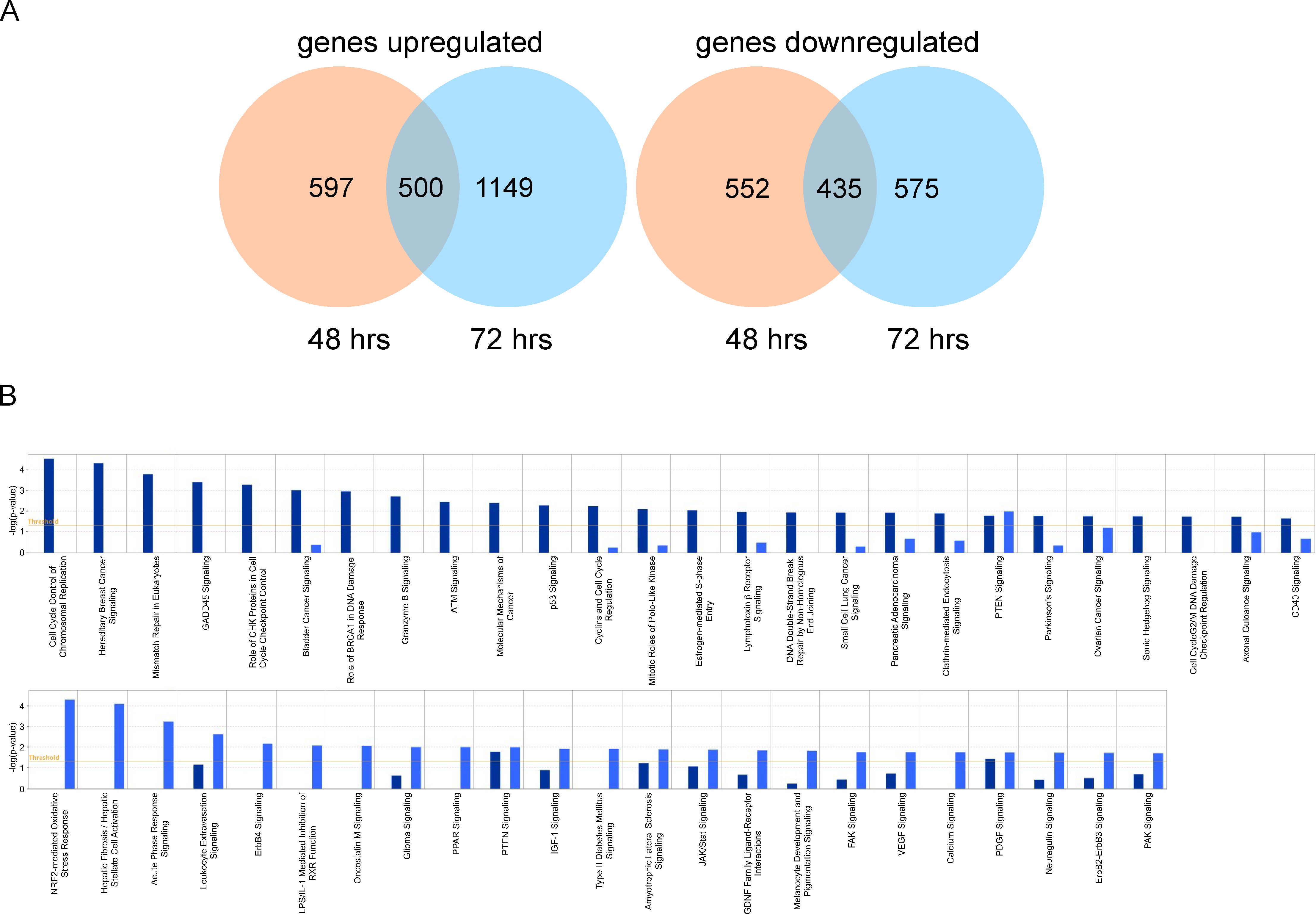

Supplement: S1 Fig — (A) Venn Diagram of genes up- and down-regulated in chick BP 48 or 72 hrs after gentamicin injection compared with control. (B) GO categories enriched in up- (top, dark blue bars) or down-regulated genes (bottom, light blue bars) using the IPA package. Mouse orthologs of differentially expressed genes in chick BP were used for analysis. (TIF) [file pone.0157768.s001.tif]

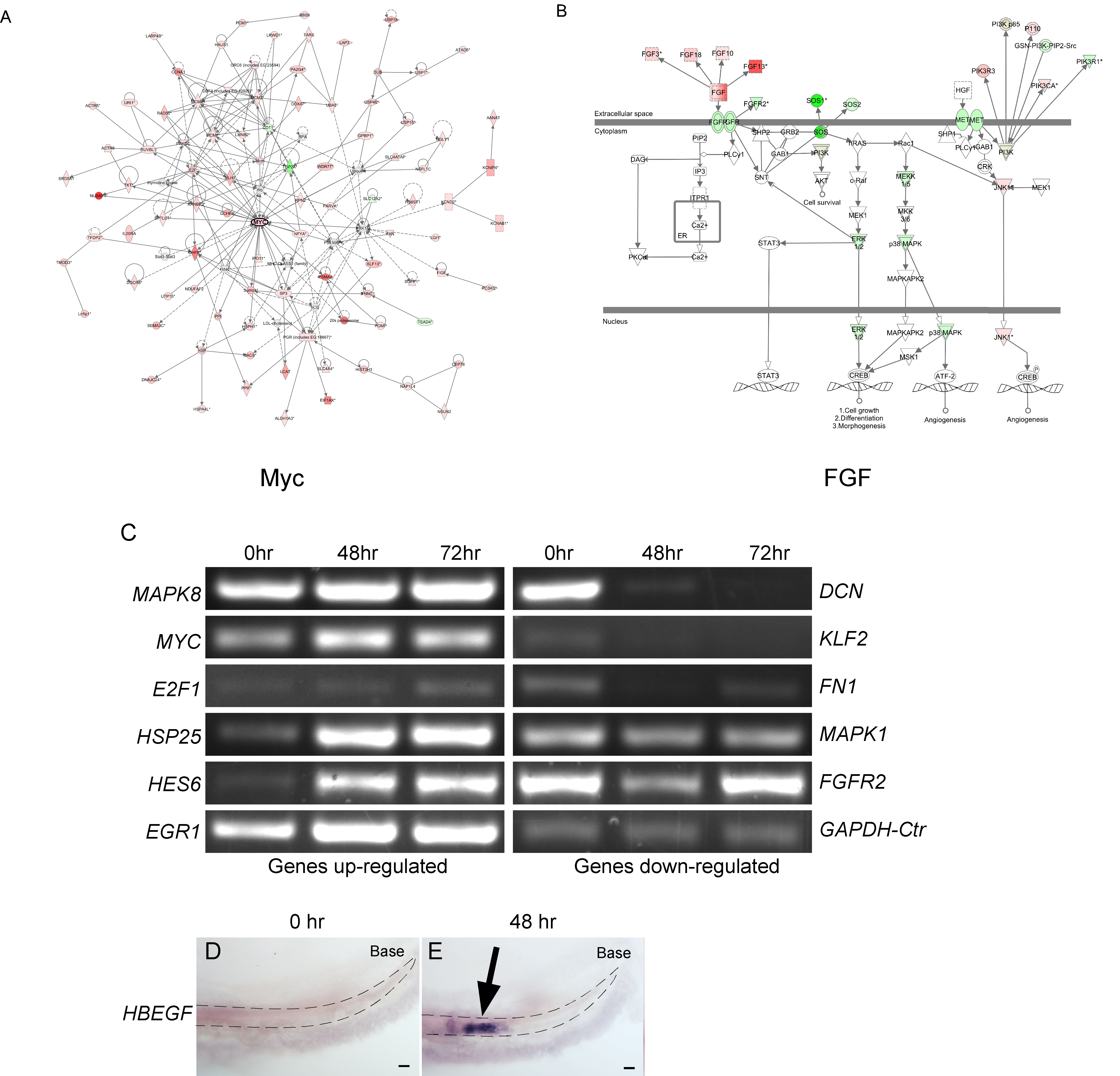

Supplement: S2 Fig — Pathways regulated by c-Myc (A) and Fgf (B) by IPA analysis. Genes up-regulated in chick BP during HC regeneration were marked in red and those down-regulated were marked in green. (A) c-Myc is a central node for the network formed by the up-regulated genes in proliferation. (B) A large number of Fgf members showed differential expression patterns during HC regeneration. (C) Semi-quantitative RT-PCR of selected genes from microarray studies showed differential expression in chick BP 48 or 72 hrs after gentamicin treatment. GAPDH is the control (Ctr). (D-E) In situ hybridization of HBEGF in the whole mount chick BP. HBEGF showed up-regulation in the sensory epithelium (marked by dotted lines) close to base (proximal end) 48 hrs after gentamicin treatment compared with control (0 hr). Scale bars: 50 μm (TIF) [file pone.0157768.s002.tif]

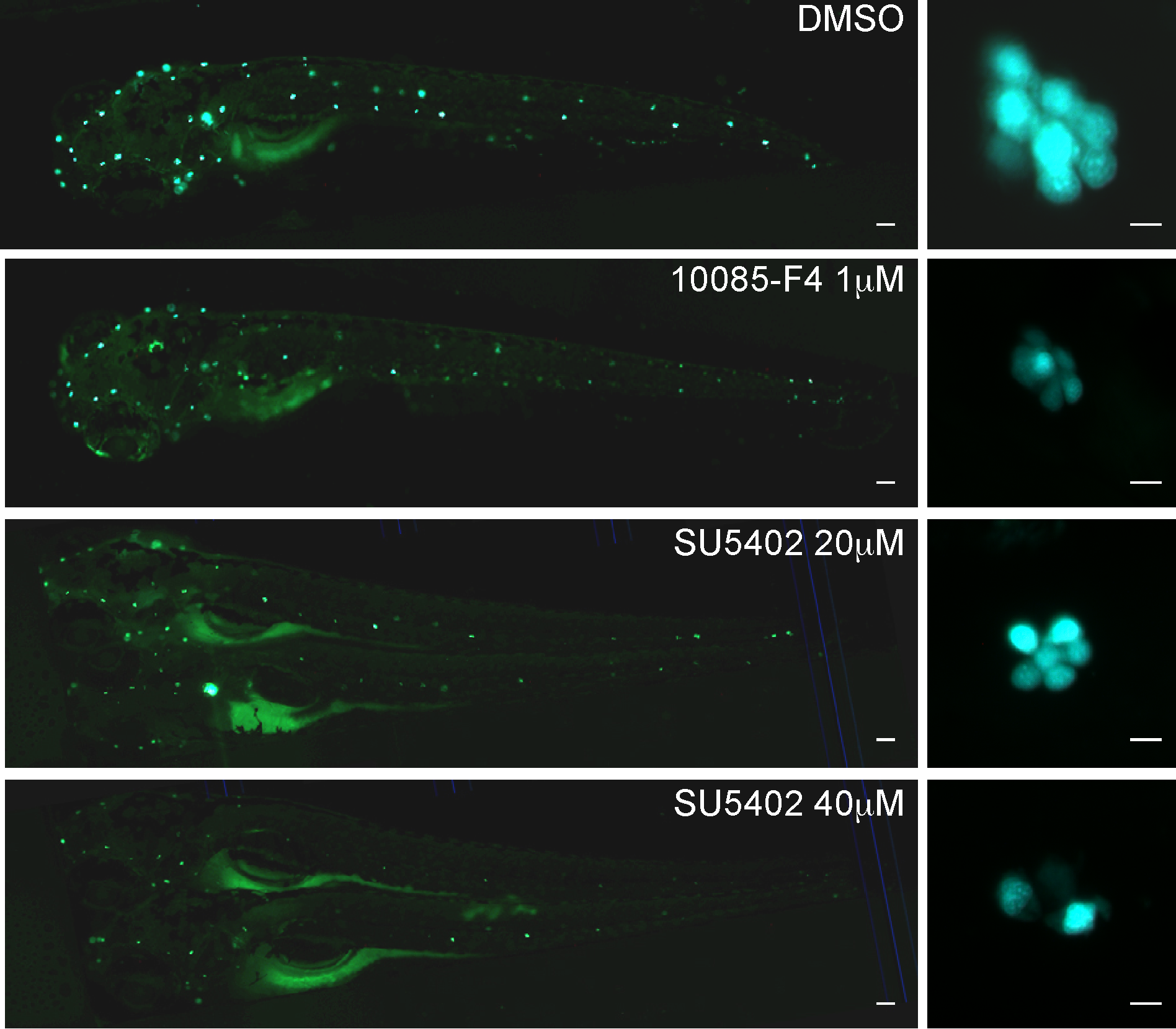

Supplement: S3 Fig — 5-dpf zebrafish larvae were treated with different inhibitors or DMSO after neomycin-induced HC death. 72 hrs later, the HCs were labeled with Yo-Pro-1. The pictures of whole fish (left) and enlarged neuromast L1 (right) showed the reduction of HC number in the inhibitor-treated neuromasts. Scale bars: left panel, 50 μm; right panel, 10 μm. (TIF) [file pone.0157768.s003.tif]

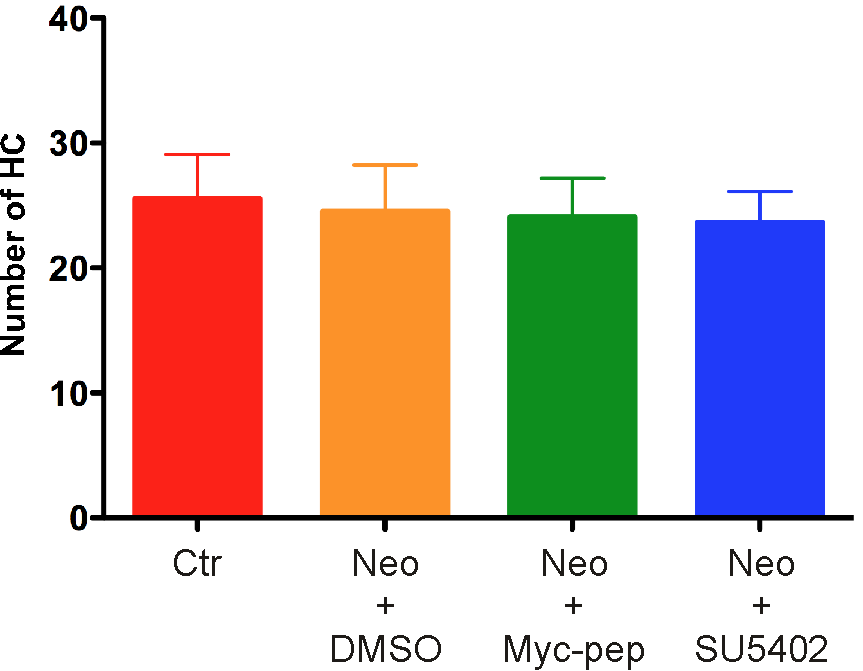

Supplement: S4 Fig — 5-dpf zebrafish larvae with neomycin treatment were treated for 72 hrs with 100 nM c-MYC inhibitor Int-H1-S6A, F8A (Myc-pep) or 20 μM SU5402, followed by replacement with fresh media for additional 72 hrs. HCs were labeled with HCS1 antibody. There was no significant difference in the number of HCs between the inhibitor-treated groups and DMSO-treated (Neo+DMSO) or no-treatment control (Ctr). (TIF) [file pone.0157768.s004.tif]

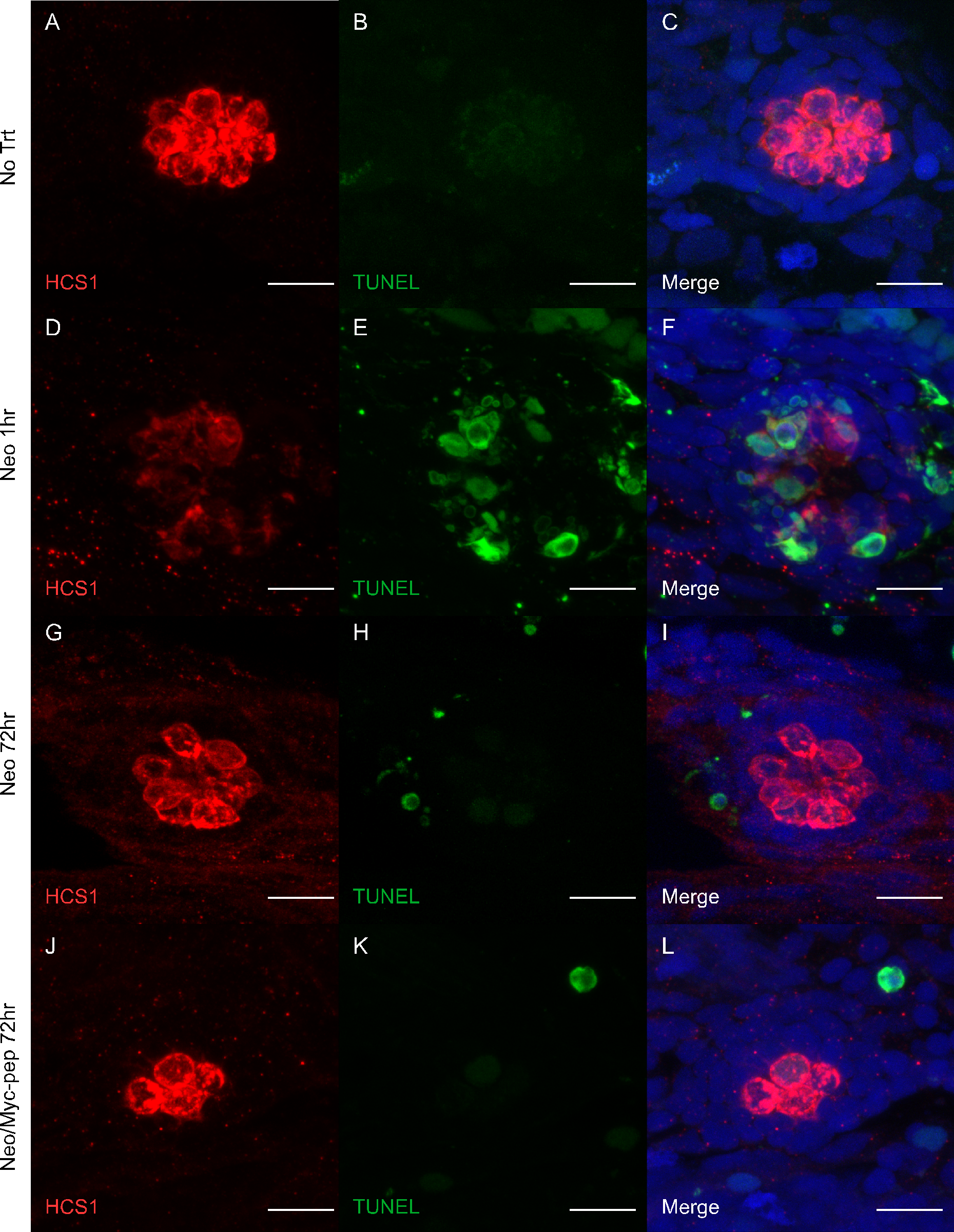

Supplement: S5 Fig — 5-dpf zebrafish larvae with neomycin treatment were then treated with or without 100 nM c-MYC inhibitor Int-H1-S6A, F8A for 72 hrs (G-I, J-L). Larvae without neomycin and inhibitor treatment (No Trt) and larvae collected 1 hr after neomycin treatment were used as controls. The fish were stained with HCS1 antibody (A,D,G,J) to label HCs and TUNEL assay (B,E,H,K) to measure apoptosis. No significant difference in apoptosis signal was observed between inhibitor-treated and non-treated fish (TUNEL+ cells per neuromast: 0.4 ± 0.2 for No Trt, n = 14; 0.4 ± 0.1 for Neo 72hr, n = 15; 0.6 ± 0.2 for Neo/Myc-pep 72hr, n = 15). All TUNEL signals were from outside of the neuromast (I,L). In the positive control (D-F), a significant increase in the TUNEL+ cells were seen inside the neuromast. Scale bars: 10 μm (TIF) [file pone.0157768.s005.tif]

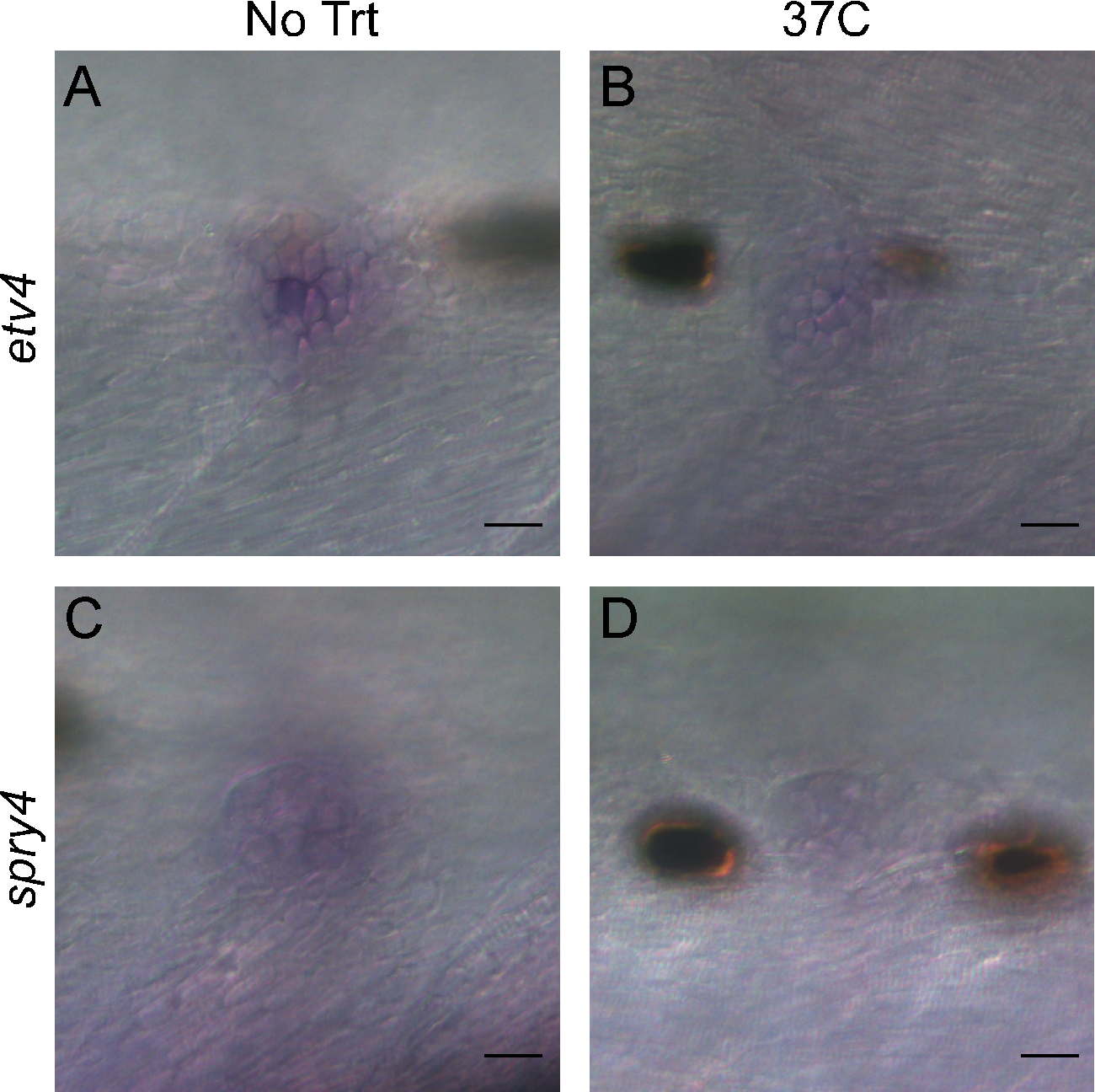

Supplement: S6 Fig — In situ hybridization showed Fgf targets etv4 (A,B) and spry4 (C,D) were relatively down-regulated in hsp70l:dn-fgfr1:GFP (Hsp) zebrafish neuromasts at 37°C compared to control. Scale bars: 10 μm (TIF) [file pone.0157768.s006.tif]

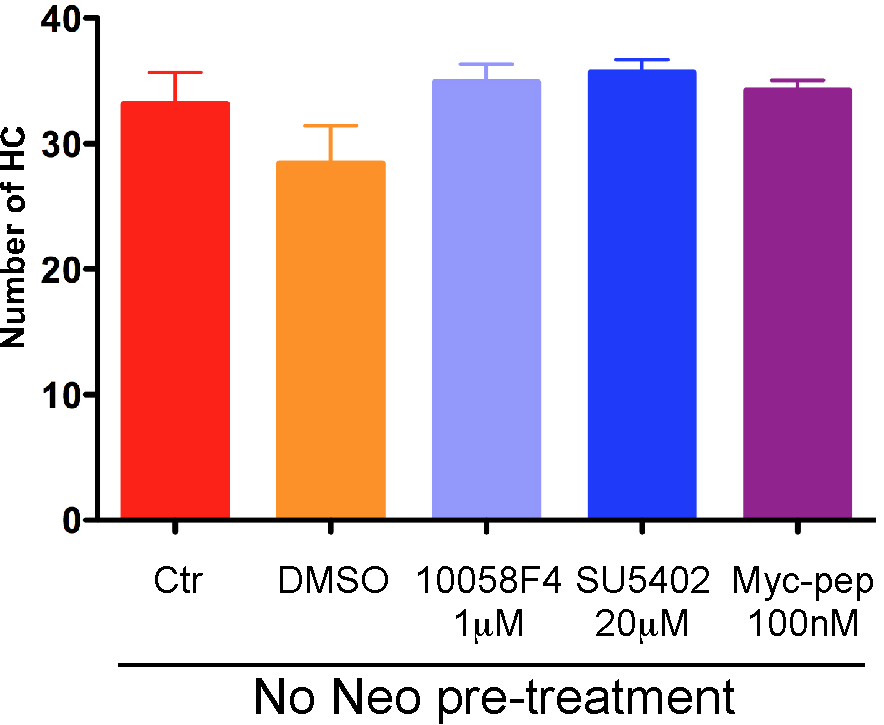

Supplement: S7 Fig — 5-dpf zebrafish larvae without neomycin treatment were treated for 72 hrs with different c-Myc and Fgf inhibitors at the highest concentrations used for our experiments. There was no significant difference in the number of HCs after the inhibitor treatment in comparison to DMSO-treated (DMSO) or no-treatment control (Ctr). (TIF) [file pone.0157768.s007.tif]

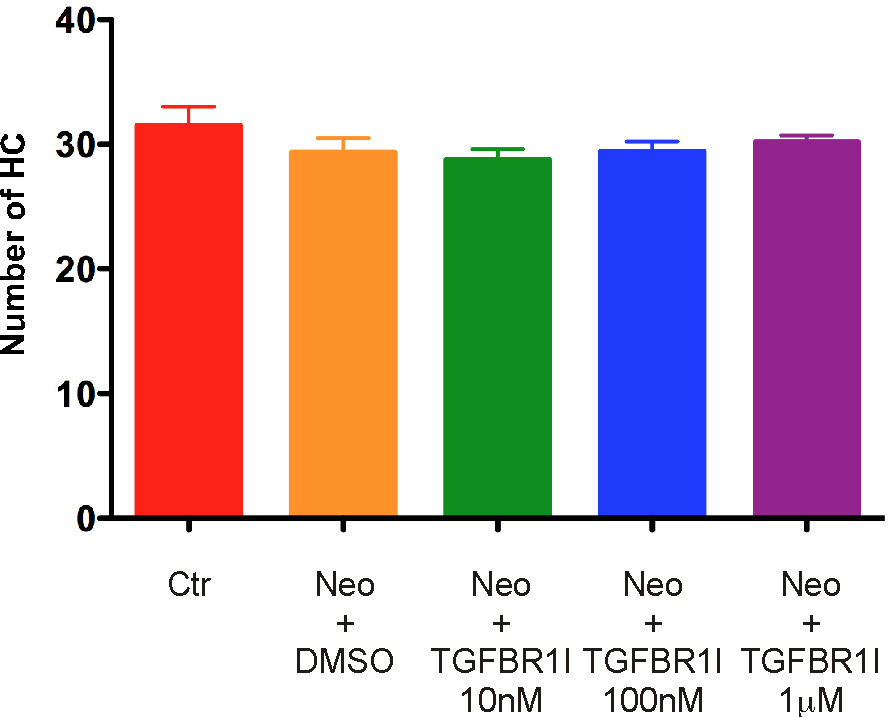

Supplement: S8 Fig — Quantification of Yo-Pro-1-labeled HCs of the 5-dpf neomycin-treated zebrafish neuromasts with different concentrations of TGFBR1I for 72 hrs showed no effect on hair cell regeneration compared to the no-treatment control (Ctr). (TIF) [file pone.0157768.s008.tif]

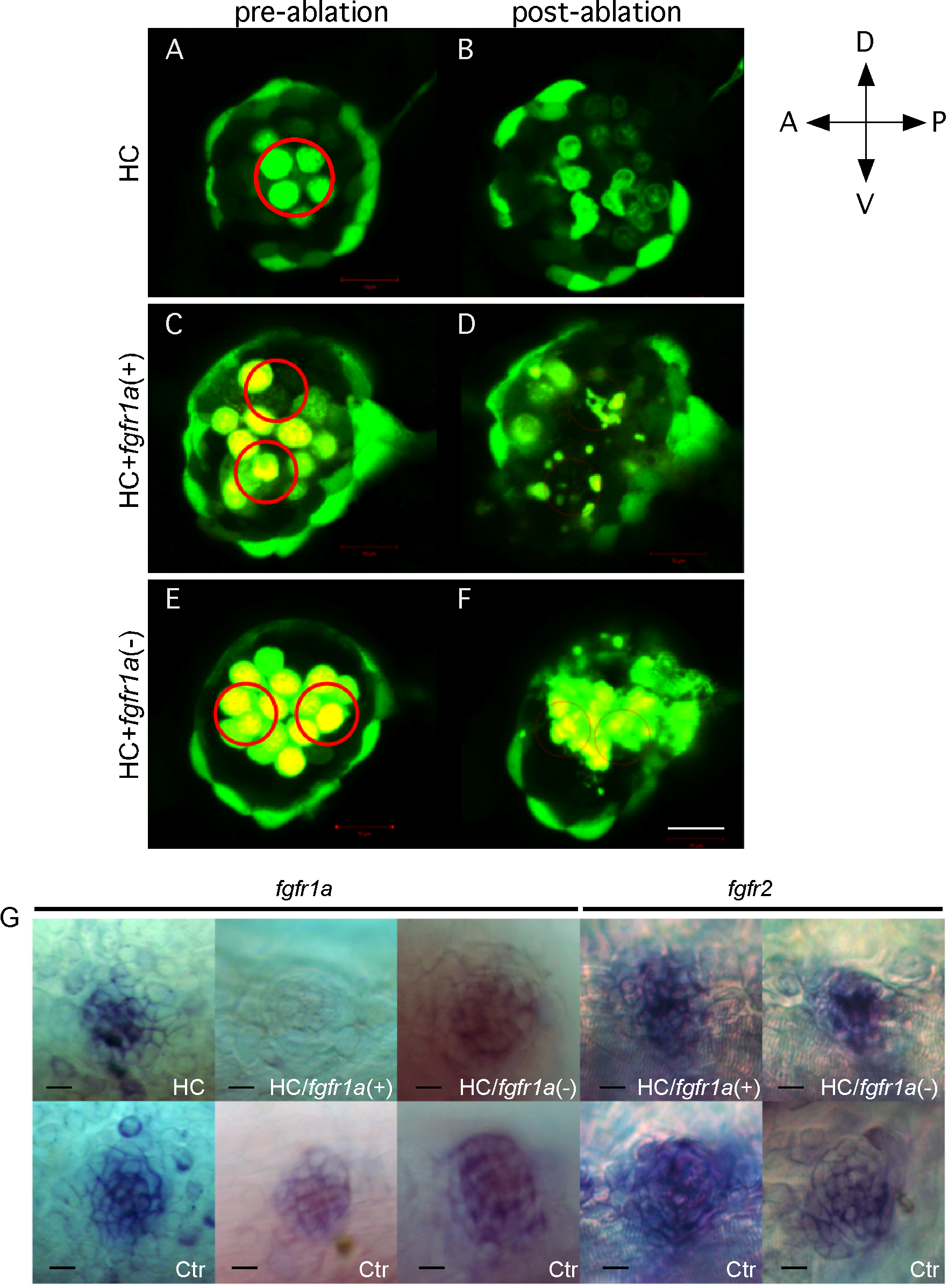

Supplement: S9 Fig — Hybrid larvae of pou4f3:GFP and ET20 fish were used to ablate HCs alone (A,B), and HC/fgfr1a(+) SCs (C-D), or HC/fgfr1a(-) SCs (E-F). Neuromasts before (A,C,E) and after ablation (B,D,F) were shown. The red circles are the target areas for ablation. A-P, anterior-posterior; D-V, dorsal-ventral. Scale bar, 10 μm. (G) In situ hybridization of fgfr1a confirmed the ablation of fgfr1a(+) cells in HC/fgfr1a(+) ablation group, where fgfr1a is undetectable; whereas in HCs or HC/fgfr1a(-) ablation group, fgfr1a signal is still present. Similar ablation did not change fgfr2 signal. Ctr, unablated neuromasts labeled with fgfr1a. Scale bars: 10 μm. (TIF) [file pone.0157768.s009.tif]
